# Examining multilevel influences on parental HPV vaccine hesitancy among multiethnic communities in Los Angeles: a qualitative analysis

**DOI:** 10.1186/s12889-023-15318-2

**Published:** 2023-03-22

**Authors:** Michelle B. Shin, Kylie E. Sloan, Bibiana Martinez, Claradina Soto, Lourdes Baezconde-Garbanati, Jennifer B. Unger, W. Martin Kast, Myles Cockburn, Jennifer Tsui

**Affiliations:** 1 Department of Child, Family, and Population Health Nursing, School of Nursing, University of Washington, Seattle, WA United States; 2 Department of Population and Public Health Sciences, Keck School of Medicine, University of Southern California, Los Angeles, CA USA; 3 Department of Molecular Microbiology & Immunology, Keck School of Medicine, University of Southern California, Los Angeles, CA USA

**Keywords:** Racial/ethnic minority communities, HPV vaccination, Vaccine hesitancy, Medical mistrust, Multilevel influences

## Abstract

**Background:**

Human papillomavirus (HPV) vaccine hesitancy is a growing concern in the United States, yet understudied among racial/ethnic minority parents. We conducted qualitative research to understand parental HPV vaccine hesitancy and inform community-specific, multilevel approaches to improve HPV vaccination among diverse populations in Los Angeles.

**Methods:**

We recruited American Indian/Alaska Native (AI/AN), Hispanic/Latino/a (HL) and Chinese parents of unvaccinated children (9–17 years) from low-HPV vaccine uptake regions in Los Angeles for virtual focus groups (FGs). FGs were conducted in English (2), Mandarin (1), and Spanish (1) between June-August 2021. One English FG was with AI/AN-identifying parents. FGs prompted discussions about vaccine knowledge, sources of information/hesitancy, logistical barriers and interpersonal, healthcare and community interactions regarding HPV vaccination. Guided by the social-ecological model, we identified multilevel emergent themes related to HPV vaccination.

**Results:**

Parents (n = 20) in all FGs reported exposure to HPV vaccine information from the internet and other sources, including in-language media (Mandarin) and health care providers (Spanish). All FGs expressed confusion around the vaccine and had encountered HPV vaccine misinformation. FGs experienced challenges navigating relationships with children, providers, and friends/family for HPV vaccine decision-making. At the community-level, historical events contributed to mistrust (e.g., forced community displacement [AI/AN]). At the societal-level, transportation, and work schedules (Spanish, AI/AN) were barriers to vaccination. Medical mistrust contributed to HPV vaccine hesitancy across the analysis levels.

**Conclusion:**

Our findings highlight the importance of multilevel influences on parental HPV vaccine hesitancy and decision-making and the need for community-specific messaging to combat medical mistrust and other barriers to HPV vaccination among racial/ethnic minority communities.

## Introduction

Safe and effective vaccines that protect against infection of high-risk human papillomavirus (HPV) types associated with cervical and other cancers have been available in the United States for nearly two decades [1]. However, adolescent HPV vaccination rates remain suboptimal. Only 62% of the adolescents aged 13–17 were up to date with HPV vaccines in the United States in 2021 [2, 3]. While national rates have been increasing, local level data show uptake in racial/ethnic minority communities have remained much lower. For example, the 2018 Los Angeles County data reported lower rates of HPV vaccine initiation among Hispanics (58%) and Asians (53%) compared to non-Hispanic White population (70%) [4]. This is especially concerning for racial/ethnic minority communities where the incidence and mortality of HPV-associated cancers are disproportionately higher [5, 6].

Vaccine hesitancy is a growing public health concern, which has been further amplified during the COVID-19 pandemic especially in diverse race/ethnic communities that are disproportionally impacted by vaccine preventable conditions like HPV-related cancers [7]. The World Health Organization’s Strategic Advisory Group of Experts on Immunization defines vaccine hesitancy as “delay in acceptance or refusal of vaccine despite availability of vaccine services”[7]. Recent literature indicates HPV vaccine hesitancy is prevalent among about one-fifth of parents [8, 9]. Our previous study showed concerns around HPV vaccine side effects and perceived newness of HPV vaccines were primary reasons for vaccine hesitancy among racial/ethnic minority parents [9]. Influences on HPV vaccine hesitancy exist at multiple levels (e.g., individual, family, and community) and sources of vaccine information are complex among racial/ethnic minority communities [10–12]. For example, parental confidence in HPV vaccine safety is related to parental demographic such education level, cultural beliefs, and exposure to media [13, 14]. Exposure to vaccine misinformation, defined as “any health-related claim of fact that is false based on current scientific consensus” [15], also may play a role in HPV vaccine hesitancy [16, 17]. However, few studies have examined the relationship between HPV vaccine hesitancy and misinformation in race/ethnic minority communities.

In addition to vaccine hesitancy, racial/ethnic minorities experience common and distinct barriers across diverse communities, emphasizing the need for culturally specific, tailored approaches. For example, many studies have found limited knowledge about HPV and HPV vaccines and cultural and linguistic barriers [18–20]. Lack of provider recommendation and access to regular sources of medical care also have been identified as barriers to HPV vaccine uptake among adolescents in Los Angeles [21–23]. While studies found that HPV or HPV vaccine knowledge is an important determinant of vaccine intention and uptake among Asian American parents [24], others found mixed association among Hispanic/Latino/a (HL) parents [25]. Several studies have noted that concerns for sexual promiscuity is a barrier to HPV vaccination among HL population [26, 27], whereas medical and vaccine mistrust has been found to be a prominent reason for not receiving HPV vaccine in the American Indian/Alaska Native (AI/AN) communities [28]. However, a few studies have examined multilevel influences on parental HPV vaccine hesitancy in the context of existing barriers and facilitators that are specific to diverse racial/ethnic minority communities.

This qualitative study explores multilevel influences on parental HPV vaccine hesitancy to further contextualize and explain findings from our prior work that demonstrated a positive association between medical mistrust and HPV vaccine hesitancy [9]. We specifically aim to examine HPV vaccine hesitancy across multiple racial/ethnic communities to understand the barriers and opportunities to inform community-specific multilevel approaches to improve HPV vaccination among diverse populations.

## Methods

### Study sample

We recruited parents or healthcare decision-makers/guardians (referred to as “parents” hereafter) with at least one child between ages 9–17 years (adolescents) who had not initiated the HPV vaccine series, including some parents with multiple children where vaccination status may differ. We purposively sampled from racial/ethnic minority communities in the greater Los Angeles region where HPV vaccinations rates are low. We partnered with two local community organizations serving racial/ethnic minority communities: The University of Southern California (USC) Leslie and William McMorrow Neighborhood Academic Initiative (NAI) and the United American Indian Involvement, Inc (UAII). NAI is an academic enrichment program that serves primarily immigrant (88% HL and 2% Asian) families in low-income neighborhoods of Central and East Los Angeles [9, 29, 30]. UAII is the largest provider of human and health services for AI/AN communities living in Los Angeles [31]. The focus group participants were recruited by language (English, Spanish, Mandarin) through community partners, who distributed in-language flyers through their member listservs to solicit participation. Information about the focus groups was also advertised at virtual parent weekend workshops hosted by NAI in May and June of 2021. Interested parents were provided a link to an online registration form in English, Spanish, and Mandarin, screened for study eligibility, and asked to provide their date/time availability and focus group language preference.

Focus groups were conducted virtually, using Zoom. Each focus group was conducted by a facilitator and a notetaker who were fluent in English and target language (Spanish or Mandarin Chinese); the AI/AN focus group was conducted by an AI/AN facilitator and an AI/AN notetaker. Focus group facilitators obtained verbal informed consent from each participant at the beginning of each session. Focus groups lasted approximately 45–60 min. All groups were audio-recorded and later transcribed verbatim and translated to English by an external translation service, frequently used for community research, if needed. The participating parents received a $50 gift card for their time. This study was approved by the USC Institutional Review Board (UP-20-00541).

### Data collection

To understand the context and processes related to HPV vaccine hesitancy, we developed a structured language-translated focus group guide that prompted discussions about the topics of sources of health information, HPV vaccine knowledge and attitudes, logistical barriers to vaccination, and interpersonal, healthcare and community interactions regarding HPV vaccination. Table 1 provides questions and prompts from the guide, which were informed by our prior work on HPV vaccine hesitancy in similar communities [9] and literature review [32–34]. The guide was translated into Spanish and Chinese by multilingual/cultural research staff and reviewed by English and Mandarin-speaking NAI staff familiar with the parent population for literacy level suitability (approximately 8th grade). The English interview guide for the AI/AN focus group was reviewed by AI/AN research team members for cultural/literacy appropriateness.

**Table 1:** Focus Group Guide by Topics

Topics	Focus Group Questions and Prompts
Health Information (Information about diseases, medications, or vaccines)	• Where do you usually get health information? o From a doctor, at a clinic, on TV, from friends and family • What would you say is the source you trust the most with information about health? o Doctor, clinic, TV, magazines
HPV vaccine knowledge	• What have you heard about the HPV vaccine? o What do you think is the purpose of the HPV vaccine? o Have you heard anything about the HPV vaccine being related to cancer? If so, what have you heard? o What do you think affects your risk for cervical cancer, oropharyngeal or other cancers related to HPV? Does your risk impact your decision about HPV vaccine?
HPV vaccine attitudes	• What are your thoughts about getting the HPV vaccine for your children? o Are there any issues that concern you about getting the HPV vaccine? o Have your thoughts on COVID-19 vaccine impacted your decision on the HPV vaccine for your children?
HPV vaccine hesitancy	• What are some of your biggest barriers to getting your child the HPV vaccine/What are the main reasons that your child has not received the HPV vaccine? o Doctor didn’t discussed it; doesn’t know if it’s safe; COVID restrictions; logistical barriers, etc. • Would you like to get more information about the HPV vaccine? o (IF YES) What kind of information about the HPV vaccine are you interested in? • What would be your preferred source for this information? o Doctors, community members, friends and family, TV, media

### Data analysis

We used the Centers for Disease Control and Prevention (CDC)’s social-ecological model for prevention to guide our analysis, which focuses on influences at multiple levels [35]. Each transcript was de-identified and coded by two team members who did not participate in the focus group. Codes were inductively based on interview guide topics and deductively derived from emerging themes and organized by levels of influence from the social-ecological model (individual, interpersonal, community and societal/structural levels). After the coding was completed, our multidisciplinary team, consisting of members with nursing science, health services, health behavior, and social work expertise, engaged in qualitative interpretive analysis. The team met in a series of four iterative meetings after the coding was completed and each immersing and reflecting on the transcripts. We compared code and had discussions to resolve discrepancies if there were any and redefined the codes if necessary. We also discussed emerging themes until saturation was reached across the focus groups, which we defined as the point in which “no new categories or themes emerge” and selected illustrative quotes at each level of influence [36, 37]. We also examined recurring themes within any single category and discussed convergence and divergence between and across the focus groups.

## Results

Four focus groups with 20 participants total were conducted between June and August 2021: two groups were in English, one in Mandarin and one in Spanish. One of the English focus groups was with AI/AN-identifying parents and the second English focus group was comprised of parents from multiple racial/ethnic groups within the NAI program. Characteristics of focus groups and the participants are described in Table 2. Most participants were female caregivers/mothers of multiple adolescents ages 9–16 years. We identified nine themes corresponding to the individual, interpersonal, community, and societal levels of the social-ecological model.

**Table 2:** Demographic Characteristic of Focus Group Participants (n = 20)

Focus Group Language	Parent Race/Ethnicity	Number of Participants	Participant Sex	Unvaccinated Adolescent Child’s Sex, Age, and Relationship to participant
Chinese	All Asian	4	1 male, 3 females	- P1:16 y/o daughter, 15 y/o son - P2: 14 y/o son - P3: 15 y/o daughter, 9 y/o son - P4: 16 y/o daughter, 15 y/o son
English	3 Hispanic/Latino/a 1 did not provide information	4	All females	- P1: 12 y/o son, 14 y/o daughter - P2: 9 y/o son - P3: 16 y/o daughter - P4: Did not provide information
Spanish	All Hispanic/Latino/a	7	All females	- P1: 16 y/o daughter - P2: 12 y/o daughter, 14 y/o daughter, 16 y/o daughter - P3: 17 y/o daughter, 15 y/o twins (son and daughter); 9 y/o daughter - P4: 14 y/o daughter, 17 y/o son - P5: 11 y/o daughter, 17 y/o daughter - P6: 17 y/o daughter - P7: 9 y/o daughter, 15 y/o daughter
English	All American Indian/Alaska Native	5	All females	- P1: 8 granddaughters (no age provided) - P2: 13 y/o, 15 y/o, 16 y/o (no sex provided) - P3: 9 y/o, 11 y/o, 16 y/o (no sex provided) - P4: 19 y/o - P5: 11 y/o

### Individual level

Two themes that contributed toward parental HPV vaccine hesitancy identified at the individual (parent) level were: confusion about the HPV vaccines leading to exposure to misinformation online and concerns about side effects/newness of the vaccine that were amplified during the COVID-19 pandemic.

#### Theme 1: Confusion about HPV vaccines lead parents to access HPV vaccine information on the internet, which expose them to HPV vaccine misinformation

All focus groups discussed using the internet as a source of health information. Some parents shared how searching the internet led to misinformation about HPV vaccines. One parent in the English focus group recounted that what they read online led to fear, and ultimately led to the decision not to vaccinate their children due to concerns about long-term effects on fertility and the chemical content of vaccines: *“Yeah, I usually just browse… And as I research online, it makes me research even more, but by the end of the night, I’m very confused. I don’t even know what to believe anymore. And that’s what it is with HPV. Initially, I wanted to go along with [the HPV vaccine] for my two kids. But as I research, I ended up not doing it.”* (Parent 1, English/mixed-ethnicity focus group). Some parents also described having to closely examine online health-related information to ascertain whether they could trust the content. Parents in the Chinese focus group described sorting through internet sources that they consider to be unreliable or untrustworthy, including exaggerated news headlines and links shared using WeChat.

All focus groups expressed confusion about the HPV vaccine, including the target age group for vaccination (Spanish focus group), which genders should receive the vaccine (English/mixed-ethnicity focus group), whether the vaccine is mandatory for school attendance (AI/AN focus group), and duration of vaccine effectiveness (AI/AN focus group). One parent in the Spanish focus group stated: *“Well, before [the providers] said [HPV vaccine is] for the women, but now, [they say] it is to prevent cancer. But in my case, I would like to [be] explain[ed], exactly what [it is] and what it’s exactly for.”* (Parent 4, Spanish focus group). Another parent in the Chinese focus group expressed a desire to have more data about the proportion of the population who is vaccinated for HPV and the HPV vaccine’s efficacy before making the decision to vaccinate their child. When the facilitator probed about the type of data, such as from the CDC and the World Health Organization, the parent answered: *“I trust more [the] numbers that are related to the Asian population, as our body types are similar.”* (Parent 3, Chinese focus group).

Parents discussed feeling motivated by the responsibility to protect their children despite feeling hesitant about HPV vaccine. As described by one AI/AN parent: *“As for me, no matter what, we could do prevent…ultimately, we don’t know [what’s] in the future. They may get an illness…no matter how much we do for our children and ourselves…So, I think in informing ourselves and just obtaining information when we can, where we can, will help us kind of guide ourselves through for our children and other kids with us.”* (Parent 3, AI/AN focus group). While the confusion about the HPV vaccine information led to hesitancy, many parents articulated the need for specific information about eligible vaccination age range, duration of vaccine efficacy, purpose of the vaccine and the desire to learn more to guide the healthcare decision-making for and with their children, further highlighting the importance of awareness and education.

#### Theme 2: Parents identified concerns about side effects and newness of the HPV vaccine as contributors to HPV vaccine hesitancy, which were amplified during the COVID-19 pandemic

Focus groups discussed concerns about potential side effects, perceived newness, and the young target age of HPV vaccines. One parent in the English focus group (mixed-ethnicity) said they were fearful of the unknown side effects: *“My daughter, she doesn’t have the [HPV] vaccine. Always, my doctor asks me [about it] when she has appointments. But I’m scared for their future. I don’t know if it’s necessary or not.”* (Parent 2, English/mixed-ethnicity focus group). Another parent in the Spanish focus group said she was initially worried that the HPV vaccine contained cancer, but ultimately learned more about it from the provider and agreed to vaccinate one of her children: *“Well, what I was worried about was, if you can get cancer [from the HPV vaccine]. So [initially] I did not agree, but the doctor instructed me to read [about the vaccine]. And then, I agreed and signed off on getting the vaccine [for my daughter].”* (Parent 7, Spanish focus group).

The perceived newness of the HPV vaccine contributed to hesitancy across all focus groups. One AI/AN parent reported relying on the “traditional” childhood vaccines (i.e., measles, mumps, and rubella) because they had been in existence for longer than the HPV vaccines. Interestingly, some parents across focus groups decided to get their children vaccinated against COVID-19 but not HPV. When discussing concerns about the newness of the HPV vaccine compared to the COVID-19 vaccines, one parent in the English group expressed confusion: *“…I think they[‘ve been] studying [the HPV vaccine] for I don’t know how many years. And they’re still studying it…so just you make me confused because two of my kids, they have [the COVID-19 vaccine already]. But my youngest one, he doesn’t have it yet so I’m, I don’t know, confused.”* (Parent 3, English/mixed-ethnicity focus group). One parent in the Chinese focus group viewed COVID-19 vaccination as more of a priority and urgent than the HPV. They said *“We should wait until she gets older”* for the HPV vaccine and then said, *“Unlike COVID-19, everyone needs to get vaccinated. The pandemic is very serious.”* (Parent 2, Chinese focus group).

Some parents discussed feeling burdened by vaccine-related decision-making for their children because they could not fully know the potential side effects and long-term consequences of the vaccines. Overall, while parents encountered conflicting online health information, expressed confusion, and shared concerns leading to their initial HPV vaccine hesitancy, parents were not outright opposed to HPV vaccination and wanted to learn more about HPV vaccines.

### Interpersonal level

At the interpersonal level, parents expressed varying opinions about involving adolescents as vaccine decision-makers. Other family members and friends acted as both barriers and facilitators of vaccination. Parental trust and prior experiences with providers/healthcare also impacted vaccine decision-making. Overall, themes at the interpersonal level revealed the complexity of relationships between parents and adolescents, other family members, and healthcare providers surrounding vaccine decision-making.

#### Theme 3: Adolescent children are involved in the vaccine decision-making in varying degrees

Some parents in AI/AN and Spanish focus groups discussed talking with their children about vaccine-related decisions; while parents in the Mandarin focus group indicated they usually make vaccine-related decisions for their children. The conversations around COVID-19 vaccines for both parents and the adolescents started discussions about other adolescent vaccines in many families, including the HPV vaccine. One parent in the Spanish focus group shared heeding to their adolescent child’s request to delay vaccination: *“I think my 14-year-old daughter [refuses to go to the vaccine appointments] because she knows the doctor’s going to draw my attention [to her weight]. That is why I have stopped [bringing up the vaccine appointments with her].”*

(Parent 4, Spanish focus group). In contrast, one parent in the Mandarin focus group stated, *“At the beginning [when the COVID-19 vaccine was approved for adolescents, my children] were hesitant, but then I pushed them to get vaccinated, because the COVID-19 is getting more serious.”* (Parent 2).

Parents in the Spanish focus group discussed the difficulty engaging adolescents in a conversation due to their developmental stage: *“Sometimes girls at a certain age do not listen to dads anymore. [They say,] ‘no, you don’t know.’…if someone else told them about it [HPV vaccine] in the schools, maybe they would be more encouraged to get the [HPV] vaccine.”* (Parent 2, Spanish focus group). This group expressed desire for more adolescent-targeted, developmentally appropriate educational materials about the HPV vaccine, and suggested it would help adolescents to receive more information about HPV vaccines directly from other sources, including school-related sources.

#### Theme 4: Family and friends function both as barriers and facilitators to HPV vaccine decision-making

Parents shared how they serve as a source of health information for vaccine decision-making for other family members and also consult them when needed. One parent from the Spanish focus group shared how HPV vaccine information they shared with the extended family was met with resistance due to concerns about sexual promiscuity: *“I have several relatives or friends who have daughters of [adolescent] age… And they do not agree to give them the [HPV] vaccine, because it is like admitting that their kids have a sex life, you know? I, myself, do not agree with that, if I can save their lives with a simple vaccine, I will do it… if that will save them from disease, of an infection, of a whatever, I agree with the vaccine…”* (Parent 2, Spanish focus group).

Parents also found consulting with their family members, especially those working in healthcare, could be helpful in making healthcare decisions. However, parents from the AI/AN, Spanish and Chinese focus groups shared how they have been more cautious about sharing and receiving health-related information with friends and family since the COVID-19 pandemic as health advice has become politicized. One AI/AN parent shared: *“There’s a lot of my friends, a lot of people I know are in the health business. So, I get a lot of information through there. But this whole COVID thing, it’s kind of hard to get information because everybody’s different. There’s no one that’s alike from the other.”* (Parent 1, AI/AN focus group) Overall, parents’ discussions with family and friends could act as barriers that contributed to their decisions to not vaccinate their children against HPV but also could serve as facilitators for parents to share or seek out additional health information about vaccines, including the HPV vaccine.

#### Theme 5: Experiences with providers/healthcare system can impact parental trust in vaccine recommendations

About half of the parents (eight of 20) across all focus groups shared that they trust health information from their providers over the internet or other sources. While parents across all language groups encountered misinformation when consulting the internet (Theme 1) or when talking to family members about health information (Theme 3), some parents reported how these more unreliable sources of information prompted useful conversations with their healthcare providers: *“[I] just Google the question I have, and then I see some different resources. Sometimes I struggle with it, so I call my doctor for any issues I have.”* (Parent 3, English/mixed-ethnicity focus group).

While many parents shared how much they trust their providers, parents were also influenced by past negative experiences with providers and healthcare systems that influenced their decision-making about vaccination for their adolescents: *“[When] my son was just a baby, I was giving him liquid albuterol [from his pediatrician]…and I noticed he was shaky and jittery…It was too much. So, I stopped giving it to him. And I stopped going to that pediatrician too. After that, I just started thinking. I need to ask more questions.”* (Parent 3, AI/AN focus group) Some parents discussed how they learned to advocate for themselves with their providers and chose to seek out more information: *“I don’t mean any disrespect – but [some doctors act] as if the doctor is doing us a favor…Instead of the doctor providing us a service that we’re paying for – we are in other words the customer. And we’re not treated like that all the time…. it’s almost like you have to take your power back as the patient that this is the service that I’m paying for, and I need quality care.”* (Parent 4, AI/AN focus group).

Overall, adolescents emerged as active participants in vaccine-related discussions and some parents requested adolescent-directed education materials about the HPV vaccine. Parents balance input from their children, other family members, and providers to navigate vaccine decision-making for their adolescents. Both positive and negative experiences with providers and healthcare system impacted trust in vaccine recommendation and HPV vaccine hesitancy.

### Community level

Themes emerging at the community level highlighted specific opportunities to address vaccine hesitancy related to community-specific historical injustices and context for medical mistrust in the HL and AI/AN communities. Each focus group specified trusted sources of information in their communities that could be leveraged for dissemination of HPV vaccine information.

#### Theme 6: Historical injustices and medical mistrust contribute to vaccine hesitancy in racial/ethnic minority communities

Some parents from the AI/AN focus group talked about the displacement of their ethnic communities, such as relocation of tribes during the 1950s (Parent 1), and how displacement continues to impact how they access healthcare. When discussing the best way to share health information with their community, one AI/AN parent shared: *“Not everyone is connected with UAII [a nonprofit organization serving the AI/AN community] or different [AI/AN] organizations…Everyone in Los Angeles County who are Native American are kind of scattered throughout the county…if you only stick to resources or agencies, you’re only going to get the people who are accessing those services. There are so many [AI/AN people] in Los Angeles County that are not accessing those services.”* (Parent 4, AI/AN focus group).

While the group was comparing newness of the HPV vaccine and the COVID-19 vaccines, one HL parent from the English/mixed-ethnicity focus group expressed fear of sterilization: *“The COVID-19 vaccine [has been around] just for a couple months. And we don’t know what the side effects are. We hear about the youngest [child] for sterilization, so we don’t know. So, I told my husband, if not mandatory, I don’t have to let [the kids] get it.”* (Parent 3). The possibility of infertility impacted the parent’s vaccine decision-making for their children regarding HPV and COVID-19 vaccines.

#### Theme 7: Community trusted sources of health information can be important avenues for dissemination of HPV vaccine information

Each focus group mentioned different sources of health information that are widely used in their communities, including in-language media, WeChat, printed and mailed information, emails, and text messages. In the Chinese focus group, in-language radio was discussed in addition to news on the internet shared by friends and family members locally and abroad via WeChat. Some parents in the Chinese focus group also mentioned that they rely on printed letters from the Los Angeles County Department of Public Health or emails and text messages from their children’s school districts to receive reliable health information. Two parents in the Chinese focus group mentioned that they only receive health information passively through in-language radio during work or emails from schools, rather than seeking information from multiple sources because of their busy work schedules. One parent also said they trust and prioritize information from the school district: *“[We rely on] information distributed by the school because they [put emphasis] on health issues. We read all the news they shared, in comparison, we only read news online occasionally.”* (Parent 4, Chinese focus group).

Some parents in the AI/AN focus group said they prefer to receive health information on printed materials from community-based organizations including UAII and Temporary Assistance for Needy Families (TANF), but also mentioned that seeking community resources has become difficult since the COVID-19 pandemic moved many services online. Other AI/AN parents mentioned text message reminders to make vaccine appointments from their providers’ office. While they thought information about HPV vaccination on social media would be helpful to share with their children and grandchildren (e.g., YouTube), they were concerned about the trustworthiness of information shared on these platforms.

Parents in AI/AN and Spanish focus groups stated that they prefer their adolescents to have health information delivered from a nurse and for their children to receive health information from their schools or from school-based health personnel: *“Ideally, in schools, there should be a qualified person who would tell the girls about that [HPV] vaccine, the risks, the benefits; in groups that are already old enough to get the [HPV] vaccine, who would prepare them and encourage them to [get the HPV vaccine], because it is for their [own] good, for your well-being, for your health.”* (Parent 2, Spanish focus group).

One parent in the AI/AN group emphasized how educating both the parents and the children, especially through schools, would be helpful for adolescents to make informed health decisions: *“There’s no nurse there [in the school-based clinics anymore]. It would be really helpful if somehow each county and/or maybe geographical area that encompassed some districts…to be able to have a nurse go in once every six months, every four months, have some type of town hall meeting for students and parents to give them the information… So, if parents are not informed with their kids, it’s going to be hard…You’re making that decision for your child. They have the right to know as well as what’s going into their body. And it’s good for them to start getting that practice so that they can know that [their] health is important.”* (Parent 4, AI/AN focus group).

### Societal/structural level

One theme that consistently emerged across focus groups in the societal level was the persistent mention of structural/logistical barriers to HPV vaccination, even among parents who are not opposed to the HPV vaccine.

#### Theme 8: Lack of transportation and appointment scheduling continue to be logistical barriers in medically underserved areas

Parents mentioned they had difficulty scheduling for HPV vaccine appointments due to their busy work schedules and lack of transportation. One parent in the Spanish focus group stated: *“When I had my youngest children… we had a lot of problems because since I did not drive and the clinic was far away, my husband had to leave work or ask for a day off to take the children to a physical check-up”* (Parent 3, Spanish focus group). Other parents expressed difficulties with making appointments for HPV vaccine due to the COVID-19 pandemic: *“Because of the pandemic I have not taken my children to physical check-ups, because there were no appointments; but [my husband and I] agree that we have to [get] our daughter [the HPV vaccine]”* (Parent 3, Spanish focus group). Overall, themes at the societal level highlighted how parents experienced structural barriers like lack of protected time from work, transportation, and limited appointments in the clinics in their areas due to the COVID-19 pandemic despite intending to vaccinate their children against HPV.

## Discussion

Our findings highlight the importance of understanding multilevel influences of parental HPV vaccine hesitancy and decision-making (as described in the social-ecological model [35]) to inform community-specific messaging to combating medical mistrust and other barriers for HPV vaccination among racial/ethnic minority communities. While parents across the focus groups shared some common barriers to HPV vaccination, we also found some distinct characteristics that should be considered in future intervention development through cultural tailoring. Specifically, we observed that many parents have encountered HPV vaccine misinformation through social networks and internet sources, which negatively impacted their understanding and decision-making for HPV vaccination for their adolescents. While other studies have examined online and social networks as sources of HPV vaccine misinformation and hesitancy [38], our study highlights community-specific channels and contents in which racial/ethnic minority parents living in low HPV vaccine uptake areas become exposed to vaccine misinformation. In the Chinese focus group, for example, language-specific media and WeChat were mentioned as sources of information. Similarly, Chong et al. found language-specific COVID-19 vaccine misinformation among Asian American subgroups on various social media platforms [39]. In addition, our findings indicate community-specific trusted sources of information that can be leveraged to disseminate HPV vaccine information to address hesitancy in racial/ethnic communities. For example, community-based organizations were emphasized as important sources of information in the AI/AN focus group, and both AI/AN and Spanish focus group expressed desire for adolescent-directed school-based resources. Other literature supports that information source characteristics and cultural adaptation of content is needed to optimize dissemination of HPV vaccine interventions in diverse ethnic communities [40–42]. However, we observed that racial/ethnic minority parents not only had preferred channels of information but relied on their trusted sources and personal relationships with health care providers and family and friends to navigate the proliferation of adolescent vaccine misinformation. These findings highlight the importance of tailoring both the content and the dissemination channels for specific communities to address HPV vaccine hesitancy effectively.

Our data also highlighted the role adolescents have in vaccine decision-making. This finding contrasts with some studies that found that adolescents rarely influence the parents’ HPV vaccine decision-making in clinic settings [43, 44]. The reason we may have observed a strong parental preference to include adolescents in decision-making could be that parents with limited English proficiency (LEP) or health literacy tend to rely on their children as language brokers [45, 46], and that our focus group captured the dynamic between the parents and their children in the milieu of their home where they feel more comfortable rather than clinic settings. It has been documented that adolescents want to learn more about the HPV vaccine [47], and the determinants of their hesitancy are poorly understood [48]. Targeted HPV vaccine interventions for LEP and marginalized communities that include adolescents in the decision-making process warrant further exploration.

Medical mistrust emerged as an important contributor to HPV vaccine hesitancy in all levels of analysis (individual, interpersonal, community and societal) across focus group, providing context for the relationship between medical mistrust and parental HPV vaccine hesitancy in our prior survey findings [9].The historical and present context of discrimination and structural racism against AI/AN, HL and Asian Americans in the medical system [49–51], and the resulting medical mistrust in these communities have been well-documented [52, 53]. Our finding that historical community context impacts parental vaccine decision-making supports other studies showing medical mistrust was consistently associated with less favorable attitudes towards the HPV vaccine and its uptake [54]. Community focused strategies and policies that acknowledge the historical and geographical context of structural racism are needed to effectively address medical mistrust in racial/ethnic minority communities and the healthcare providers that serve them [55].

Logistical barriers to HPV vaccination in the United States have been well-documented in prior studies, including appointment scheduling, transportation, cost barriers, and social determinants of health such as race, nativity and socioeconomic status that lead to overall health care access [56–59]. Parents in our study mentioned persistent structural barriers to HPV vaccine uptake that extend beyond vaccine awareness, hesitancy, or misinformation as barriers to accessing HPV vaccines. Pandemic-related challenges such as limited catch-up appointments in the clinics were also noted. School-based interventions may address the logistical barriers in communities where the social needs are greater [60]. For example, others have found that parents who struggle to take off work and uninsured parents are more willing to partake in school-based vaccination programs [61, 62].

### Limitations

Some limitations to this study should be noted. First, we recruited parents purposively from communities and geographies in Los Angeles with low HPV vaccine uptake to conduct virtual focus groups during the pandemic. While all parents reported having at least one unvaccinated HPV vaccine eligible child and shared many similarities (e.g., neighborhood of residence, low acculturation level), our four focus groups consisted of a smaller than average number of participants (3–7 parents per group) compared to pre-pandemic periods, limiting the potential generalizability of focus group discussions to the broader communities of interest. For example, participants in our study were parents who were able to engage virtually and had available time on a weeknight or weekend to engage in conversation about adolescent vaccines. Another limitation of the small focus group sizes is the generalizability of our findings to race/ethnic minority populations. We conducted our analysis by language groups rather than race/ethnicity to match our recruitment method and follow the lead of our community partners who are experts in communicating with diverse parents. The literature on vaccine hesitancy related to HPV and other vaccines also supports that there are differences within those who share race/ethnicity by other factors such as primary language, acculturation, and urbanicity [63–65]. While comparing the association between vaccine hesitancy and other factors such as race/ethnicity and primary language is beyond the scope of this qualitative analysis, further research will be needed to inform intervention tailoring. Despite the small focus group sizes, multiple themes converged across the groups, adding to the evidence that saturation can be reached despite small sample sizes of 4–8 focus groups [66]. Lastly, each focus group had different facilitators to accommodate different language groups, which may have introduced slight variations in discussion focus across the groups. To ensure consistency, our research team met regularly throughout the interview process and used a pre-determined, structured focus group guide.

## Conclusion

There is a need to optimize community-specific messaging in combating medical mistrust and overcoming HPV vaccine hesitancy as well as continuing to address the structural barriers for HPV vaccination that are persistent among racial/ethnic minority communities. Gains in HPV vaccination rates were severely disrupted during the COVID-19 pandemic, leading to substantial missed doses among adolescents [67]. Findings from this study can inform messaging for HPV vaccinations at the adolescent, parent, provider, and community levels to help counter and confront an opposing and controversial movement against HPV and other vaccinations. Given the multilevel barriers to HPV vaccination faced by racial/ethnic minority communities, future public health efforts must address HPV vaccine misinformation, medical mistrust that stems from historical injustices, and logistical barriers to healthcare access by disseminating HPV vaccination information through community-specific messaging and trusted sources of information and integrating social care into healthcare delivery to increase adolescent HPV vaccination in communities with low uptake.

## Data Availability

The datasets generated and analyzed during the current study are not publicly available to protect the participants’ privacy. De-identified data from this study will be made available on case-by-case basis (as allowable according to institutional IRB standards) from the corresponding author on reasonable request.

## Abbreviations

AI: American Indian
AN: Alaska Native
CDC: Centers for Disease Control and Prevention
HPV: Human Papillomavirus
HL: Hispanic/Latino/a
LEP: Limited English Proficiency
NAI: Neighborhood Academic Initiative
TANF: Temporary Assistance for Needy Families
UAII: United American Indian Involvement, Inc
USC: University of Southern California
